# Treatment recommendations for psoriatic arthritis

**DOI:** 10.1136/ard.2008.094946

**Published:** 2008-10-24

**Authors:** C T Ritchlin, A Kavanaugh, D D Gladman, P J Mease, P Helliwell, W-H Boehncke, K de Vlam, D Fiorentino, O FitzGerald, A B Gottlieb, N J McHugh, P Nash, A A Qureshi, E R Soriano, W J Taylor, for the Group for Research and Assessment of Psoriasis and Psoriatic Arthritis (GRAPPA)

**Affiliations:** 1 University of Rochester Medical Center, Rochester, New York, USA; 2 University of California at San Diego, San Diego, California, USA; 3 University of Toronto, Toronto, Ontario, Canada; 4 Seattle Rheumatology Associates, Seattle, Washington, USA; 5 Academic Unit of Musculoskeletal and Rehabilitation Medicine, University of Leeds, Leeds, UK; 6 Johann Wolfgang Goethe University, Frankfurt, Germany; 7 University Hospitals Leuven, Leuven, Belgium; 8 Stanford University, Stanford, California, USA; 9 St. Vincent’s University Hospital, Dublin, Ireland; 10 Tufts Medical Center, Boston, Massachusetts, USA; 11 Royal National Hospital for Rheumatic Diseases, Bath, UK; 12 University of Queensland, Cotton Tree, Queensland, Australia; 13 Harvard Medical School, Boston, Massachusetts, USA; 14 Hospital Italiano de Buenos Aires, Buenos Aires, Argentina; 15 University of Otago/Wellington, Wellington, New Zealand

## Abstract

**Objective:**

To develop comprehensive recommendations for the treatment of the various clinical manifestations of psoriatic arthritis (PsA) based on evidence obtained from a systematic review of the literature and from consensus opinion.

**Methods:**

Formal literature reviews of treatment for the most significant discrete clinical manifestations of PsA (skin and nails, peripheral arthritis, axial disease, dactylitis and enthesitis) were performed and published by members of the Group for Research and Assessment of Psoriasis and Psoriatic Arthritis (GRAPPA). Treatment recommendations were drafted for each of the clinical manifestations by rheumatologists, dermatologists and PsA patients based on the literature reviews and consensus opinion. The level of agreement for the individual treatment recommendations among GRAPPA members was assessed with an online questionnaire.

**Results:**

Treatment recommendations were developed for peripheral arthritis, axial disease, psoriasis, nail disease, dactylitis and enthesitis in the setting of PsA. In rotal, 19 recommendations were drafted, and over 80% agreement was obtained on 16 of them. In addition, a grid that factors disease severity into each of the different disease manifestations was developed to help the clinician with treatment decisions for the individual patient from an evidenced-based perspective.

**Conclusions:**

Treatment recommendations for the cardinal physical manifestations of PsA were developed based on a literature review and consensus between rheumatologists and dermatologists. In addition, a grid was established to assist in therapeutic reasoning and decision making for individual patients. It is anticipated that periodic updates will take place using this framework as new data become available.

The articular and dermatological manifestations associated with psoriatic arthritis (PsA) are remarkably heterogeneous in the extent and type of tissue involvement. Patients with PsA, a chronic systemic inflammatory disorder, may develop not only peripheral arthritis but also axial disease, dactylitis, enthesitis and skin and nail psoriasis, with consequent adverse impact on function and quality of life (QoL).^1^,^2^ Heterogeneity is observed not only in disease manifestations but also in severity and course, which can vary from very mild psoriasis or enthesitis to widespread psoriatic plaques, disfiguring nail disease and severe joint inflammation with destruction that can result in disability and increased mortality.^3^,^4^ Moreover, comorbidities associated with psoriasis such as the metabolic syndrome can contribute to damage in multiple end-organs and often leads to markedly impaired QoL as well as early mortality.^5^,^6^,^7^

Recent progress in understanding the immunopathogenesis of PsA has been accompanied by treatment advances that have accelerated rapidly over the last decade.^8^ Despite these advances, therapeutic decisions for an individual patient with PsA can be challenging due to the diversity of clinical characteristics and the simultaneous involvement of multiple different tissues, often with varying degrees of severity. To address the need for evidence-based treatment recommendations and assist the practitioner, members of the Group for Research and Assessment of Psoriasis and Psoriatic Arthritis (GRAPPA) published systematic reviews of the literature to identify the best available evidence regarding treatment of the various manifestations of PsA.^1^,^9^ Herein, we present treatment recommendations that were formulated by rheumatologists and dermatologists in GRAPPA in conjunction with PsA patients, based on evidence from these systematic reviews and consensus opinion. These recommendations were developed to provide the best care for patients with PsA, regardless of economic or political considerations.

## Methods

**Figure 1:**
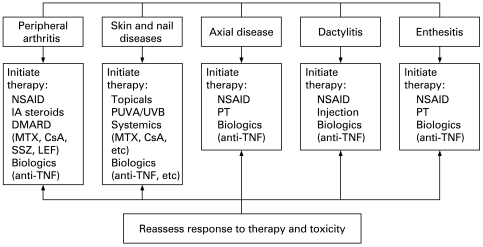
Group for Research and Assessment of Psoriasis and Psoriatic Arthritis (GRAPPA) treatment guidelines for psoriatic arthritis, categorised by disease characteristics and distinct organ involvement. Anti-TNF, anti-tumour necrosis factor; CsA, ciclosporin A; DMARD, disease-modifying antirheumatic drug; IA, intra-articular; LEF, leflunomide; MTX, methotrexate; NSAID, non-steroidal anti-inflammatory drug; PT, physiotherapy; PUVA, psoralen–ultraviolet light A; SSZ, sulfasalazine; UVB, ultraviolet light B. Reproduced with permission from Kavanaugh *et al*.^1^

The target audience for these treatment recommendations is all clinicians who care for PsA patients. First, formal literature reviews were performed by members of GRAPPA. To capture data regarding the varied areas of involvement characteristic of PsA, articles were selected that provided evidence supporting the treatment of peripheral arthritis, spinal disease, skin and nail disease, enthesitis and dactylitis in the setting of PsA (fig 1). These articles were reviewed and graded, and the results have been published.^10^,^11^,^12^,^13^,^14^,^15^,^16^ The evidence was graded using the approach of the Institute of Medicine.^17^ Wherever possible, effect sizes were calculated to quantify the extent of efficacy or toxicity. Effect size is the mean difference in effect between treatment and control, divided by the standard deviation of the difference.^18^ Effect sizes of 0.2 or less are considered small and unimportant in terms of efficacy, whereas effect sizes greater than 0.8 are considered large and suggest high efficacy.

Reviewers graded the evidence and treatment recommendations for PsA in accordance with recommendations from the Agency for Health Care Policy Research (AHCPR), as shown in table 1.

**Table 1:** Grading of evidence sources and recommendations

Evidence or recommendation	Grade
Evidence source as recommended by the Agency for Health Care Policy Research (AHCPR):	
Meta-analysis of randomised controlled trials (RCT)	1a
One or more RCT	1b
One or more controlled trials (without randomisation)	2a
Other well designed studies (quasiexperimental)	2b
Non-experimental studies (descriptive studies such as comparative or correlation studies, or case-control studies)	3
Expert committee opinions, clinical experience	4
Preliminary recommendations for treatment of psoriatic arthritis (using the best available evidence extracted from published literature):	
Category 1 evidence	A
Category 2 evidence, or extrapolation from category 1 evidence	B
Category 3 evidence, or extrapolation from category 1 or 2 evidence	C
Category 4 evidence or extrapolation from category 2 or 3 evidence	D

To address the nuanced and complex application of the results of these studies to the heterogeneous situations that arise in the clinic, focus groups were assembled, comprising experts in rheumatology and dermatology with specific experience in the care of PsA and psoriasis and patients with PsA. Next, subcommittees were formed for each of the five domains, and recommendations were drafted based on evidence and consensus between rheumatologists, dermatologists and patients for psoriasis, peripheral arthritis, axial disease, dactylitis and enthesitis. Each of these subcommittees developed a definition of mild, moderate, or severe for their individual domains. Finally, the subcommittee chairs met with AK and CTR (authors) to refine the recommendations (mild–moderate–severe categories) in each domain. The recommendations for each domain and the grid with the three categories were voted on by the entire membership of GRAPPA. Note that the grid was designed as a tool to assist in making treatment decisions for individual patients and as such is largely based on expertise. Data that became available after the systematic reviews were published were also considered by the various subcommittees.

A total of 19 items covering the diagnosis, assessment and treatment of the 5 PsA clinical manifestations were submitted for a vote using a web-based interface (Survey Monkey; http://www.surveymonkey.com/). Each item was rated on a 5-point scale (1 = strongly agree to 5 = strongly disagree). The Disagreement Index (DI) was derived from the 30th and 70th percentile of the respondents’ ratings, adjusted for symmetry between the central point of the interpercentile range and the mid-point of the rating scale. The adjustment factor was derived from experimental work that compared different definitions of what constituted “disagreement” among panels of various sizes. The number of respondents, percentage respondents in the 30th and 70th percentile, percentage respondents in category 1 or 2, and the mean and standard deviation were calculated.

## Results

The results of the survey are shown in the supplementary material. In all, 70 rheumatologists and dermatologists responded to the questionnaire. For 16 of the 19 items, 80% of the respondents agreed or strongly agreed. The three areas where agreement was not as strong included the use of the Bath Ankylosing Spondylitis Disability Activity Index (BASDAI) to measure axial disease activity over time (75.7%) and to assess axial treatment response (78.7%) and the algorithm for the treatment of psoriasis (69.2%). The strength of each recommendation (grades A–D) is included.

### Peripheral arthritis

#### Diagnosis and assessment

Diagnosis of PsA should follow the CASPAR (for Classification Criteria for Psoriatic Arthritis) criteria.^19^ We consider inflammation to include such features as pain involving joints, spine and/or enthuses associated with erythaema, warmth and swelling; prominent morning and rest stiffness.

Preferably, diagnosis of psoriasis should be confirmed by a dermatologist and inflammatory musculoskeletal disease by a rheumatologist, or either one by an appropriately qualified health professional.

Baseline evaluations of PsA should include the following domains (consensus on core set of domains for psoriatic arthritis assessment established at the eighth Outcome Measures in Rheumatology (OMERACT 8) conference, 2006).^20^

- Peripheral joint assessment (68 joints for tenderness; 66 joints for swelling).
- Pain (patient-reported on a visual analogue or category rating scale).
- Patient global assessment of disease activity.
- Physical function (eg, as measured by the Health Assessment Questionnaire (HAQ)).
- Health-related QoL, as assessed by a general measure (eg, Short Form 36 (SF-36)) or a PsA-specific measure (eg, Psoriatic Arthritis QoL (PsAQoL)).
- Fatigue, measured by patient self-report or a general instrument (eg, Functional Assessment of Chronic Illness Therapy (FACIT)).
- Acute phase reactants (eg, C-reactive protein (CRP) or erythrocyte sedimentation rate (ESR)).

Radiographic assessment is encouraged according to clinical manifestation and doctor discretionary judgment.

Factors associated with a poor prognosis related to the progression of peripheral joint disease and damage in patients with PsA include: the number of actively inflamed joints (ie, polyarticular disease, as opposed to monoarticular disease); elevated ESR; failure of previous medication trials; the presence of damage, either clinically or on *x* ray;^21^ a loss of function as assessed by HAQ; and diminished QoL as assessed by SF-36, Dermatology Life Quality Index (DLQI), or PsAQoL.

A patient should be considered a treatment failure when in spite of therapy for a length of time appropriate to the pharmacokinetic/pharmacodynamic profile of the individual agent at an appropriate dose, the patient fails to demonstrate acceptable clinical improvement. Response to treatment of peripheral arthritis in patients with PsA may be assessed using criteria initially developed for rheumatoid arthritis (RA), such as the 28-joint Disease Activity Score (DAS28, shown to be reliable and discriminative in PsA, even though it uses only 28 joints) and the European League Against Rheumatism (EULAR) response criteria, which categorise levels of disease and changes to assess response. The American College of Rheumatology (ACR) percentage response criteria (eg, ACR20/50/70) may also be used in PsA.^22^ In a recent analysis of PsA and RA outpatient cohorts,^23^ the utility of the DAS28 for PsA was questioned with regard to its applicability in settings outside of clinical trials, where patients receive therapies of varying efficacy. Response may also be considered inadequate if there is evidence of progression of joint damage on radiographs.

#### Treatment

Treatment recommendations for peripheral arthritis include non-steroidal anti-inflammatory drugs (NSAIDs), intra-articular glucocorticoid injections, disease-modifying antirheumatic drugs (DMARDs) and TNF inhibitors (see table 2).

**Table 2:** Treatment recommendations

	Disease status	Treatment recommendation	Level of evidence*	Level of agreement†	Comments
Peripheral arthritis	Mild	NSAIDs	A	90.9%	For control of joint but not skin symptoms
	NA	Intra-articular glucocorticoid injections	D		May be given judiciously to treat persistently inflamed joints, if care is taken to avoid injection through psoriatic plaques. Injections to any one joint should be repeated with caution according to clinical judgment
	Moderate or severe	DMARDs (specific recommendations follow):			For all patients with severe or moderate peripheral arthritis. Consider for mild disease if patients do not respond to NSAIDS or intra-articular steroids. No evidence supporting DMARDs ahead of TNF inhibitors, although the effect size for TNF inhibitors is much larger than that for traditional DMARDs
		SulfasalazineLeflunomideMethotrexateCiclosporine	AABB		
	Moderate or severe	TNF inhibitors	A		For patients who fail to respond to at least one DMARD therapy. The three currently available TNF inhibitors (etanercept, infliximab and adalimumab) are equally effective for the treatment of peripheral arthritis and for the inhibition of radiographic progression. Patients with poor prognosis could be considered for TNF inhibitors even if they have not failed a standard DMARD
Skin disease	Moderate to severe	Phototherapy	A	69.2%	First-line therapies:Phototherapy includes UVB/nbUVB, oral PUVA, bath PUVA, with or without acitretin. An initial trial of phototherapy should be made, unless it is not appropriate or if psoriasis is in areas that preclude phototherapy (ie, scalp, groin, axilla). All forms of phototherapy are considered as a group, although many consider that PUVA therapy carries increased risk of skin cancer compared with other UV modalities. Aggressive immunosuppression should not follow extensive phototherapy (especially PUVA), given the increased risk of melanoma and non-melanoma skin cancer in this scenario
		Methotrexate	A		
		Fumaric acid esters	A		
		TNF inhibitors	A		TNF inhibitors include etanercept, adalimumab and infliximab
		Efalizumab	A		
		Ciclosporine	A		Ciclosporine should be limited to less than 12 consecutive months because cumulative toxicity (ie, multiple courses) is not well studied
		Acitretin	A		Second-line therapies
		Alefacept	A		
		Sulfasalazine	A		Third-line therapies
		Hydroxyurea	C		
		Leflunomide	A		
		Mycophenolate mofetil	C		
		Thioguanine	C		
Nail disease	NA	Retinoids	C	69.2%	
		Oral PUVA	C		
		Ciclosporine	C		
		TNF inhibitors	C		TNF inhibitors include infliximab and alefacept
Spinal disease	Mild to moderate	NSAIDs	A	86.4%	For patients who fail therapies for mild to moderate disease
		Physiotherapy	A		
		Education, analgesia and injection of sacroiliac joint	A		
	Moderate to severe	TNF inhibitors	A		Infliximab, etanercept and adalimumab have all demonstrated efficacy in AS; the consensus was that similar treatment responses reported in AS were also likely to be observed in axial PsA
Enthesitis	Mild	NSAIDS, physical therapy, corticosteroids	D	87.9%	
	Moderate	DMARDs	D		
	Severe	TNF inhibitors	A		Evidence has been demonstrated for infliximab or for etanercept (in spondyloarthropathies)
Dactylitis	NA	NSAIDs	D	90.2%	Usually employed initially
	NA	Corticosteroids	D		Many clinicians rapidly progress to injected steroids
	Resistant	DMARDs	D		Nearly always in the context of co-existing active disease
	NA	Infliximab	A		Some evidence available

*See Methods section of manuscript for description of categories and levels of evidence.

†Percentage of survey responders who agreed or strongly agreed (see supplementary material).

AS, ankylosing spondylitis; DMARD, disease-modifying antirheumatic drug; NA, not applicable or not specifically defined; NSAID, non-steroidal anti-inflammatory drug; PsA, psoriatic arthritis; PUVA, psoralen–ultraviolet light; TNF, tumour necrosis factor; UVB, ultraviolet B light.

Systemic corticosteroids are not typically recommended in the treatment of psoriasis and are only advisable in discrete circumstances and not for chronic use, due to the potential to cause post-steroid psoriasis flare and other adverse effects (D). Gold salts, chloroquine and hydroxychloroquine also are not recommended for use in PsA.

DMARDs have the potential to reduce or prevent joint damage and preserve joint integrity and function (although none have been shown to do this in PsA). Many factors influence the choice of DMARD for the individual patient: its relative efficacy, convenience of administration, requirements of the monitoring program, costs of the medication and monitoring (including doctor visits and laboratory costs), time until expected benefit and the frequency and potential seriousness of adverse reactions. Input from a rheumatologist is often essential when initiating DMARD therapy.

A patient should be considered a DMARD failure if at least one DMARD has been failed individually or in combination in an adequate therapeutic trial, defined as treatment for ⩾3 months, of which ⩾2 months is at standard target dose (unless significant intolerance or toxicity limits the dose). Intolerance/toxicity is defined as treatment for <2 months, where treatment is withdrawn because of drug intolerance or toxicity. When treatment is withdrawn because of intolerance or toxicity after >2 months therapy, at least 2 months should have been at therapeutic doses.

Although there is no evidence for the use of combination therapy, a combination of two or more agents could be used in those patients who fail to respond to a single agent, or who present joint damage progression in spite of treatment.

### Axial disease

#### Diagnosis and assessment (based on ankylosing spondylitis (AS) criteria)

Diagnosis of axial disease should be based on the presence of two of three of the following criteria:

- Inflammatory back pain (features including onset age <45 years, symptoms >3 months, morning stiffness >30 min, insidious onset, improved with exercise, alternating buttock pain).
- Limitation of motion of cervical, thoracic, or lumbar spine in saggital and frontal planes; noted differences from AS include less pain, less limitation in movement and less symmetry. The International Spondyloarthritis Interrater Reliability Exercise (INSPIRE) has shown assessments of spinal disease in AS are also reliable in axial PsA.^24^
- Radiological criteria (eg, plain *x* ray (unilateral sacroiliitis grade 2 or more), syndesmophytes, MRI changes in sacroiliac joints of bone marrow oedema, erosions, joint space narrowing). Criteria modified from AS based on data from Helliwell *et al*.^25^

Based on experience in AS, disease activity in the spine can be reliably measured by the BASDAI, where active disease has been defined by a BASDAI score ⩾4. The BASDAI can be used to measure disease activity over time; assessment should take place after 6 weeks of treatment. A treatment response, based on definition of response in AS, is defined as a BASDAI score<3 or a reduction by 2 points.

#### Treatment

See table 2 for treatment recommendations based on evidence derived in AS. Traditional oral DMARDs such as methotrexate, leflunomide and sulfasalazine, have not been shown to be effective in axial manifestations of AS^26^ and by extrapolation, they are not considered to be of adequate efficacy for PsA axial disease, until further data are available.

### Enthesitis

#### Diagnosis and assessment

The diagnosis of enthesitis is challenging; currently three approaches have been described: clinical examination, including pain/tenderness/swelling at tendon, ligament or capsule insertion site by palpation and pressure; ultrasound with power Doppler; and MRI.

Several instruments proposed for clinical assessment of enthesitis have been tested in the INSPIRE study and were reliable in AS and PsA, but no single instrument has gained widespread acceptance.^24^ Another assessment modality is the visual analogue pain scale.

#### Treatment

See table 2 for treatment recommendations for mild, moderate, or severe enthesitis.

### Skin and nails

#### Diagnosis and assessment (for selection of topical vs systemic therapy)

For patients with mild psoriasis, candidates for topical therapy alone must meet all of the following criteria: generally asymptomatic; minimal impact on QoL; amenable and responsive to localised therapy; less than 5% for plaque psoriasis; and no incapacity and/or disability. For patients with moderate to severe psoriasis, candidates for an addition or change to systemic and/or phototherapy should meet at least one of the following criteria: symptomatic (ie, pain, bleeding, itching); more than minimal impact on QoL; inadequate response to localised therapy; body surface area generally greater than 5% for plaque psoriasis; patients with guttate, erythrodermic, or pustular psoriasis; psoriasis in vulnerable areas (face, genitals, hands/feet, nails, scalp, or intertriginous areas); or varying degrees of incapacity and disability from psoriasis.

#### Treatment

See table 2 for a list of first-line, second-line and third-line systemic therapies for treatment of psoriasis.

It is important to note that the clinician should consider all agents in a treatment level before proceeding to “lower” level. Situations rendering a specific therapy as “not appropriate” include lack of response, adverse events and poor access to therapy.

Unusual clinical subsets of psoriasis can co-occur with arthritis; thus, treatment may vary from that used in psoriasis vulgaris. For erythrodermic/generalised pustular psoriasis, consider acitretin as first-line therapy, although more research is needed. For palmoplantar pustuolosis, acitretin and oral PUVA appear to result in improvement (no evidence for either one being superior), with combination of the two providing superior response. Ciclosporine and tetracycline appear to be of modest benefit. No strict recommendations can be given, due to lack of definition of treatment response, lack of controlled studies in this area.^27^ For treatment of hand/foot psoriasis, consider topical PUVA, soriatane, efalizumab as preferable first-line agents although more research is needed in this area.

All three TNF inhibitors have shown efficacy in phase 3 randomised controlled trials, but no head-to-head trials have been published to directly compare efficacy and safety. Some data, however, suggest that etanercept may not be as effective in patients with high BMIs.^15^ In psoriasis studies, etanercept efficacy was dose-dependent, with doses as high as 100 mg per week (double the typical dose for RA and PsA patients) providing the most benefit.^28^

The efficacy of therapies for psoriatic nail disease is not well studied; see table 2 for existing evidence.^29^ Specific recommendations cannot be made due to size of studies and lack of appropriate controls.

At this time, no recommendation can be given regarding efficacy and side effect profiles of systemic corticosteroids because clinical trial data are not available. In general, monotherapy with systemic corticosteroids is to be avoided in psoriasis because skin disease can flare during or after taper. Further studies, however, are needed to evaluate the role of short-term corticosteroids in severe, pustular and erythrodermic psoriasis.

### Dactylitis

#### Diagnosis and assessment

Dactylitis, defined as uniform swelling of a digit, is due to synovitis, tenosynovitis and enthesitis together with soft-tissue oedema. Dactylitis occurs in 16–48% of cases of PsA, and acute dactylitis has been shown to be a clinical indicator of disease severity in PsA.^30^ Conversely, chronic, non-tender diffuse dactylitic swelling may be less clinically significant, although MRI appearances differ only quantitatively from acute dactylitis. Recurrent isolated dactylitis, often in the same digit(s), may be the only clinical manifestation of PsA.

#### Treatment

The treatment of dactylitis is largely empirical. See table 2 for treatment recommendations of initial and more resistant cases.

### Severity assessment in PsA

Patients may be roughly stratified in categories of “mild”, “moderate”, or “severe” for peripheral arthritis, skin disease, spinal disease, enthesitis and dactylitis according to presence of criteria noted in table 3. This table is designed to be used as a tool to assist in decision making, and rigorous adherence to the proposed stratification is not appropriate. Until numeric thresholds for mild, moderate and severe for the various instruments are validated, doctor judgment is required to appropriately stratify individual patients. Some patients may have multiple manifestations, and treatment decisions may be determined by the most severe clinical presentation. The synergistic impact of multiple simultaneous manifestations may be assessed with the patient global assessment, HAQ and disease-specific instruments (DLQI, PsAQoL). Two case descriptions are provided in table 4.

**Table 3:** Disease severity

	Mild	Moderate	Severe
Peripheral arthritis	<5 jointsNo damage on *x* rayNo LOFQoL minimal impactPt. evaluation mild	⩾5 joints (S or T)Damage on *x* rayIR to mild RxMod LOFMod impact on QoLPt. evaluation moderate	⩾5 joints (S or T)Severe damage on *x* rayIR to mild–moderate RxSevere LOFSevere impact on QoLPt. evaluation severe
Skin disease	BSA<5, PASI<5, asymptomatic *	Non-response to topicals, DLQI, PASI<10†	BSA>10, DLQI>10PASI>10
Spinal disease	Mild painNo loss of function	Loss of function or BASDAI>4†	Failure of response
Enthesitis	1–2 sitesNo loss of function	>2 sites or loss of function	Loss of function or>2 sites and failure of response *
Dactylitis	Pain absent to mildNormal function	Erosive disease or functional loss	Failure of response

*See case 1 in table 4; †see case 2 in table 4.

S, swollen; T, tender; LOF, loss of physical function; IR, inadequate response; BSA, body surface area; BASDAI, Bath Ankylosing Spondylitis Disability Activity Index; PASI, Psoriasis Activity Severity Score; QoL, quality of life; DLQI, Dermatology Life Quality Index.

**Table 4:** Case descriptions

Case	History/symptoms	Recommendation(s)
1	19-year-old male student:History of psoriasis.Presented with disabling bilateral Achilles tendonitis and right plantar fasciitis.Unable to bear weight.Initial treatment (without sustained relief) included two different NSAIDs, a 10-day course of oral corticosteroids, physiotherapy and plantar fascia injection.Symptoms have been present for 10 weeks.Mild scalp psoriasis that is well controlled with topical agents.	This patient has severe enthesitis and mild skin disease (see table 3), and he has failed therapies for mild and moderate enthesitis; a TNF inhibitor should be considered.
2	34-year-old male:Moderate to severe psoriasis since childhood.2-year history of inflammatory back pain with unilateral grade 2 sacroiliitis on a plain film of the AP pelvis; his BASDAI is 5.6.Used topical agents and phototherapy for psoriasis; has been treated with two different NSAIDS and an exercise program with no change in the BASDAI.No loss of function but mild impairment in QoL.Percentage of BSA with plaque is 5%, which is having a significant negative impact on QoL, more than the back pain.DLQI is 7.2.	This patient has moderate axial disease and moderate skin involvement (see table 3). For his axial disease, it is recommended that he have education, analgesia and sacroiliac injection. For his skin disease, a systemic agent is warranted. If the combination of axial and skin disease is severely impairing QoL and/or function, a TNF inhibitor may be considered

## Discussion

The array of disease manifestations coupled with the wide range in disease severity and course observed in PsA present formidable challenges to the treating clinician. Therapeutic decisions must be based on thorough assessments of the different areas of involvement including the skin and nails. Of note, the cumulative negative impact of widespread inflammation at various sites can be multiplicative, leading to profound impairment of patient quality of life and function. Further complicating treatment decisions are the paucity of adequately powered placebo-controlled clinical trial data for some of the most common agents used in the treatment of PsA, most notably methotrexate. Certainly, the development of improved trial design that incorporates relevant and measurable outcomes favours biological agents since many of the older studies conducted on DMARDs are under-powered and suffer design flaws. Lastly, management of PsA often requires input from rheumatology and dermatology: any treatment recommendations must be developed based on input from groups of doctors as well as their patients.^31^,^32^

The GRAPPA organisation was founded with the mission to improve the care of PsA patients based on input from all doctors and health professionals who care for PsA patients.^1^ The GRAPPA Treatment Recommendations Committee received expert opinion from over 16 dermatologists; indeed, the psoriasis treatment section of this paper was developed entirely by these doctors. Of note, however, these dermatologists focused on the treatment of psoriasis in the setting of PsA; therefore, their recommendations should not be extrapolated to psoriasis alone. The section on psoriasis treatment was also broadened to include some unusual subsets that can be particularly vexing for the treating doctor with regard to diagnosis and management.

The therapeutic strategies outlined in this manuscript represent the first treatment recommendations based on a thorough review of the literature, followed by a consensus exercise among international dermatology and rheumatology experts who care for PsA patients. Input from PsA patients provided the doctors with a deeper understanding and appreciation of how treatment options and decisions are viewed by individuals who suffer from this disease. The weaknesses of these recommendations centre primarily on the lack of studies with high levels of evidence. It should be noted that many of the agents mentioned in these guidelines are not necessarily approved by appropriate regulatory agencies for these indications; for example, fumarates are neither US Federal Drug Authority (FDA) nor European Medicines Agency (EMEA) approved (they are approved only in Germany) for the therapy of plaque psoriasis, but there is sufficient high-grade evidence to warrant their inclusion as a first-line therapy. In addition, while the sample size of participating GRAPPA members seems robust, this number represents less than half of the registered members in the organisation. Although patient-reported outcomes (physical function, QoL and fatigue) have been measured and have shown positive results, particularly with TNF inhibitors, they were not the focus of our guidelines, except as part of composite scores (eg, the HAQ and patient global assessment in the ACR scoring system). Finally, considerable differences persist among the members regarding how to assess severity for the various manifestations of PsA. While the group did have over 80% agreement regarding the content of the disease grid (table 3), it was with the understanding that this tool represents a starting point that will be modified as new trial data are published. The core domains and instruments for use in clinical trials and in the care of PsA patients have been identified by GRAPPA, and preliminary validation was obtained through the OMERACT process.^33^,^34^,^35^

Assessment and treatment of axial manifestations of PsA is very challenging because of the paucity of data. By consensus, it was agreed that ASAS guidelines be used.^36^ The cut-off point for the definition of moderate to severe disease activity in AS was chosen by ASAS to be a BASDAI score ⩾4. For BASDAI response criteria, a 50% relative change or absolute change of 2 (0–10) with expert opinion of significant improvement was chosen. However, this cut-off was formally validated by Cohen *et al*^37^ and Pavy *et al*^38^ and represents an appropriate criterion to borrow from AS for axial disease in PsA until further testing can be performed prospectively in clinical trials.

Recent studies have shown that dermatologists and rheumatologists can assess skin and joint disease with a surprising degree of agreement and accuracy.^39^ Similar studies have been published for axial disease.^24^ Efforts to develop instruments for assessment of dactylitis and enthesitis are underway, and it is anticipated that these will be tested for validity in the near future.^24^,^40^ Ultimately, a composite assessment tool that can be applied in the office setting will allow clinicians to formulate more informed treatment decisions for individual patients.

Interest in PsA has greatly intensified over the past several years due to several factors including a better understanding of disease mechanisms, improved clinical trial design and perhaps most importantly, the arrival of effective and relatively safe biological agents that have dramatically altered the treatment paradigm. Currently, a host of new treatments are in the pipeline, many of which will offer new and possibly less expensive therapeutic options. It is anticipated that the treatment recommendations outlined in this study will be refined and serve as a template for the development of revised PsA treatment updates as new data are released.
